# A prediction model for acute kidney injury in adult patients with hemophagocytic lymphohistiocytosis

**DOI:** 10.3389/fimmu.2022.987916

**Published:** 2022-09-20

**Authors:** Siwen Wang, Lichuan Yang, Jiaojiao Zhou, Jia Yang, Xin Wang, Xuelian Chen, Ling Ji

**Affiliations:** ^1^ Department of Nephrology, West China Hospital Sichuan University, Chengdu, China; ^2^ Department of Occupational Disease and Toxicosis/Nephrology, West China Fourth Hospital Sichuan University, Chengdu, China; ^3^ Department of Ultrasound, West China Hospital Sichuan University, Chengdu, China; ^4^ Department of Pediatric Nephrology, West China Second Hospital Sichuan University, Chengdu, China

**Keywords:** acute kidney injury, hemophagocytic lymphohistiocytosis (HLH), risk factors, prediction model, risk assessment

## Abstract

**Background and aims:**

Hemophagocytic lymphohistiocytosis is a clinical syndrome resulting from abnormally active immune cells and a cytokine storm, with the accompanying phagocytosis of blood cells. Patients with hemophagocytic lymphohistiocytosis often suffer acute kidney injury during hospitalization, which usually signifies poor prognosis. We would like to establish a prediction model for the occurrence of acute kidney injury in adult patients with hemophagocytic lymphohistiocytosis for risk stratification.

**Method:**

We extracted the electronic medical records of patients diagnosed with hemophagocytic lymphohistiocytosis during hospitalization from January 2009 to July 2019. The observation indicator is the occurrence of acute kidney injury within 28 days of hospitalization. LASSO regression was used to screen variables and modeling was performed by COX regression.

**Results:**

In the present study, 136 (22.7%) patients suffered from acute kidney injury within 28 days of hospitalization. The prediction model consisted of 11 variables, including vasopressor, mechanical ventilation, disseminated intravascular coagulation, admission heart rate, hemoglobin, baseline cystatin C, phosphorus, total bilirubin, lactic dehydrogenase, prothrombin time, and procalcitonin. The risk of acute kidney injury can be assessed by the sum of the scores of each parameter on the nomogram. For the development and validation groups, the area under the receiver operating characteristic curve was 0.760 and 0.820, and the C-index was 0.743 and 0.810, respectively.

**Conclusion:**

We performed a risk prediction model for the development of acute kidney injury in patients with hemophagocytic lymphohistiocytosis, which may help physicians to evaluate the risk of acute kidney injury and prevent its occurrence.

## Introduction

Hemophagocytic lymphohistiocytosis (HLH) is a life-threatening syndrome caused by an abnormally active immune system. The major players involved are immunocytes such as cytotoxic T lymphocytes and natural killer cells, as well as inflammatory factors, including interferon-γ (IFN-γ), interleukin (IL)-1β, and IL-18. Hemophagocytosis is an important feature of HLH. Primary HLH is caused by genetic defect, while secondary HLH is mainly triggered by malignant tumors, infections, and immune factors (1, 2). The detection of hemophagocytes in the bone marrow is a characteristic of HLH, but not specific, and it is common in other tissues (such as liver, spleen, lymph nodes, etc) of critically ill patients (3). HLH is clinically difficult to diagnose, progresses rapidly, and is susceptible to multi-organ failure, including kidney failure. Clinicians face great challenges in the diagnosis and treatment of HLH and the management of its complications.

Acute kidney injury (AKI) is a clinical syndrome with a rapid increase in serum creatinine and/or a rapid decrease in urine output within a short period of time (4). AKI is common in hospitalized patients and has a high mortality rate. AKI, often secondary to extra-renal events, is an increasingly common problem for physicians and surgeons. In addition, the present view suggests that AKI promotes the development of chronic kidney disease and is a risk factor for cardiovascular disease (5). However, most patients with AKI have no noticeable clinical manifestations and AKI often co-exists with other syndromes, making it easy for clinicians to overlook it, leading to delay in diagnosis and treatment.

In recent years, an increasing number of researchers have focused on renal diseases complicated by HLH, especially AKI in HLH. Some scholars observed intraglomerular hemophagocytosis and erythrophagocytic macrophages in the tubular lumen (6, 7). However, the knowledge and expertise of researchers and clinicians on AKI in HLH are still far from enough. The AKI caused by HLH is mostly reported in some cases, which presented pathologic and speculated about the cause of AKI (7, 8). At present only one retrospective cohort study in this field analyzed the characteristics of AKI, kidney disease outcome and factors of mortality. And the study showed a high incidence of AKI in the course of HLH of up to 62% and the mortality risk is significantly higher in patients combined with AKI (9). AKI in HLH diminishes patients’ quality of life and is strongly associated with high mortality. Therefore, there is a strong need to develop an appropriate model to predict AKI in patients with HLH to facilitate disease management by clinicians.

In the present study, we aimed to leverage available data to identify risk factors for AKI in HLH patients, establish a predictive model and evaluate the model to elucidate its accuracy and clinical practicality.

## Materials and methods

### Study population

**Figure 1:**
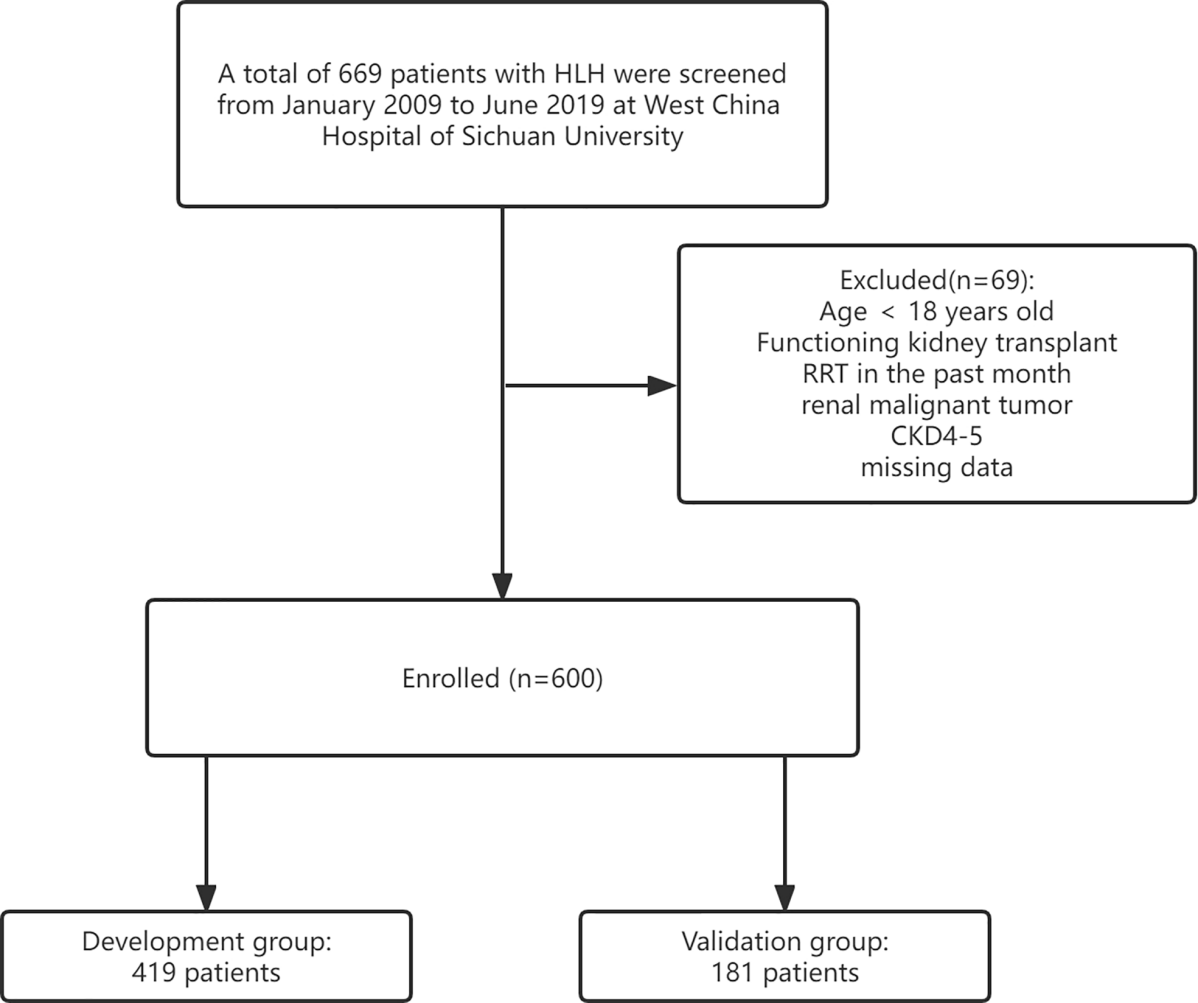
Research flowchart.

We first filtered out 669 patients diagnosed with HLH at West China Hospital of Sichuan University from January 2009 to July 2019. Patients with HLH who fulfilled the diagnostic criteria proposed by the Histocyte Society in 2004 were then enrolled (10). Patients were required to fulfill 5 of the following 8 criteria: 1) fever; 2) splenomegaly; 3) cytopenia affecting at least 2 of 3 lineages in the peripheral blood; 4) hypertriglyceridemia (triglyceride ≥3.0 mmol/L) and/or hypofibrinogenemia (fibrinogen ≤1.5 g/L); 5) hemophagocytosis in bone marrow or spleen or lymph nodes; 6) low or absent NK-cell activity; 7) ferritin ≥500 ng/ml; and 8) soluble CD25 (i.e., soluble IL-2 receptor) ≥2,400 U/ml. We did ‘ t adopt gene standard in the diagnosis of HLH because genetic testing is not generally recommended in adult patients (11). The exclusion criteria include the following: patients younger than 18 years old, kidney transplant recipients, dialysis within the past 1 month, kidney malignancy, chronic kidney disease stage 4-5 and missing data. A total of 669 individuals were included in this study, and after the screening process, we analyzed 600 patients who met the requirements. Patients were split randomly into development and validation groups in a 7:3 ratio, with 419 individuals in the development group and 181 in the validation group (**Figure 1**). The outcome indicator of interest is whether the patient experienced AKI from the start of admission to the 28th day of hospitalization.

### Clinical and biochemical data acquisition

We retrospectively gathered patients’ information from the electronic medical record database established at our hospital. Demographic characteristics included gender, age, and history of tobacco and alcohol use. Treatment modalities consisted of therapy for HLH and some supportive therapy. The main triggers of HLH were infection, tumor, and autoimmune disease (1). If a specific trigger of HLH cannot be found, it is defined as “Undetermined”. We also collected some comorbidities such as hypertension, diabetes mellitus, and heart failure. HLH-related clinical manifestations like fever, hepatosplenomegaly, and lymph node enlargement were also potential variables for our study. Laboratory based data were platelets, white blood cells, hemoglobin, baseline creatinine, baseline cystatin C, bilirubin, albumin, triglycerides, cholesterol, fibrinogen, etc. The lowest creatinine of patients within 2 days before admission was used as the baseline creatinine. If creatinine within 2 days before hospital admission was not available, the first creatinine tested after admission would be considered as the baseline creatinine.

### Definitions employed

The updates made to the organization Kidney Disease Improving Global Outcomes (KDIGO)’s diagnostic criteria in 2012 defined AKI as an increase in the serum creatinine of ≥ 0.3 mg/dl (≥ 26.5μmol/l) within 48 h, ≥ 1.5 times the baseline within the previous 7 days, or a urine volume ≤ 0.5 ml/kg/h for 6 h. Since it was not convenient to obtain the patients’ urine volume, AKI was diagnosed on the basis of the creatinine level change (12). Fever was measured as axillary temperature of 37.3°C or higher. Physical Examination and imaging were used to evaluate whether the patient had enlarged lymph nodes and hepatosplenomegaly. The recognition of edema is mainly depending on clinical signs. Vasopressors included norepinephrine, dopamine, epinephrine, phenylephrine, and vasopressin.

### Statistical analysis

This analysis of data was performed using R software (version 4.0.3; The R Foundation for Statistical Computing), and P<0.05 was considered statistically significant for this study. Continuous variables were expressed as mean ± standard deviation, and categorical variables were expressed as frequencies and percentages. Independent samples t-test (normal distribution for continuous variables), Wilcoxon rank-sum test (skewed distribution for continuous variables), and Pearson chi-square test (categorical variables) were used to determine statistical differences between the AKI and non-AKI groups, respectively.

Because of the large number of predictor variables in this study, it is appropriate to adopt least absolute shrinkage and selection operator (LASSO) regression screening variables (glmnet package) in the development group. LASSO regression filters lambda by Ten‐fold cross‐validation (13). The initial screening variables after lasso regression were screened for colinearity before modeling, and the exclusion criteria were those with variance inflation factor (VIF) values ≥ 5. Cox regression was then applied to build the model and regression coefficients, standard error values, hazard ratio (HR), 95% confidence intervals (CI) and p-values were calculated for each variable in the model. For clinical convenience, we plotted nomogram following the relative weights of the variables in the model. According to the magnitude of the regression coefficients of each influence factor in the model, a score is assigned to each value level of each influence factor, and then the individual scores are summed to obtain the total score.

The area under the receiver operating characteristic (ROC) curve (AUC) and the concordance index (Harrell’s concordance index/C-index) were used for model discrimination evaluation in the development and validation groups. Calculation of integrated discrimination improvement (IDI) to represent the role of baseline cystatin C in the model.

## Results

### Baseline characteristics of patients with HLH

During the 28 days of hospitalization, 136 (22.7%) patients developed AKI. Baseline characteristics of patients were shown in **Table 1**, including demographics, laboratory data, treatment regimens and clinical manifestations. There were 266 (57.33%) males in the non-AKI group and 79 (58.09%) males in the AKI group. The mean age of patients in the two groups was 41.56 ± 16.49 and 44.75 ± 17.30, respectively. Baseline creatinine was 69.12 ± 41.47 and 57.83 ± 23.12 umol/L, and baseline cystatin C was 1.57 ± 0.71 and 1.34 ± 0.46 mg/L for patients in the AKI and non-AKI groups, respectively.

**Table 1:** Baseline characteristics of the AKI and non-AKI groups.

Variables	Non-AKI n = 464	AKI n = 136	P
AKI stage I		50 (36.76%)	
AKI stage II		44 (32.35%)	
AKI stage III		42 (30.88%)	
Age	41.56 ± 16.49	44.75 ± 17.30	0.051
Gender			0.875
Male (%)	266 (57.33%)	79 (58.09%)	
Female (%)	198 (42.67%)	57 (41.91%)	
Tobacco use (%)	141 (30.39%)	35 (25.74%)	0.295
Alcohol (%)	118 (25.43%)	40 (29.41%)	0.354
Glucocorticoid (%)	417 (89.87%)	122 (89.71%)	0.955
Etoposide (%)	215 (46.34%)	52 (38.24%)	0.095
Nephrotoxic drugs (%)	228 (49.14%)	67 (49.26%)	0.979
Chemotherapy (%)	136 (29.31%)	37 (27.21%)	0.634
Immunosuppressant (%)	159 (34.27%)	37 (27.21%)	0.123
Vasopressor (%)	84 (18.10%)	73 (53.68%)	<0.001
Mechanical ventilation (%)	44 (9.48%)	54 (39.71%)	<0.001
monoclonal antibodies (%)	32 (6.90%)	9 (6.62%)	0.91
Triggers of HLH			
Infection (%)	227 (48.92%)	60 (44.12%)	0.324
Tumor (%)	242 (52.16%)	74 (54.41%)	0.643
Autoimmune disease (%)	25 (5.39%)	9 (6.62%)	0.585
Undetermined (%)	21 (4.53%)	15 (11.03%)	0.005
≥2 causes (%)	61 (13.15%)	12 (8.82%)	0.175
Disease history			
Tuberculous (%)	11 (2.37%)	3 (2.21%)	0.911
Tumor lysis syndrome (%)	3 (0.65%)	8 (5.88%)	<0.001
Hypertension (%)	28 (6.03%)	14 (10.29%)	0.087
Diabetes (%)	16 (3.45%)	5 (3.68%)	0.899
Heart failure (%)	21 (4.53%)	20 (14.71%)	<0.001
Viral hepatitis (%)	51 (10.99%)	13 (9.56%)	0.634
Clinical manifestations of HLH			
Lymph nodes enlargement (%)	271 (58.41%)	85 (62.50%)	0.393
Splenomegaly (%)	347 (74.78%)	96 (70.59%)	0.328
Hepatomegaly (%)	164 (35.34%)	44 (32.35%)	0.519
Fever (%)	446 (96.12%)	133 (97.79%)	0.350
Edema (%)	165 (35.56%)	69 (50.74%)	0.001
DIC (%)	66 (14.22%)	46 (33.82%)	<0.001
Maximum body temperature during hospitalization (°C)	39.32 ± 1.00	39.44 ± 0.91	0.197
Admission heart rate (Times /min)	98.02 ± 18.11	104.60 ± 18.15	<0.001
Blood pressure			
Systolic pressure (mmHg)	110.97 ± 15.95	113.17 ± 16.10	0.16
Diastolic pressure (mmHg)	68.61 ± 11.79	70.63 ± 12.92	0.086
PLT (×10^9^/L)	27.39 ± 33.41	17.14 ± 20.39	0.002
HGB (g/L)	65.14 ± 19.62	55.88 ± 14.66	<0.001
WBC (×10^9^/L)	1.43 ± 1.77	1.42 ± 1.58	0.528
Baseline Scr (umol/L)	57.83 ± 23.12	69.12 ± 41.47	0.004
Baseline Cystatin C (mg/L)	1.34 ± 0.46	1.57 ± 0.71	<0.001
Calcium (mmol/L)	1.76 ± 0.20	1.68 ± 0.23	<0.001
Phosphorus (mmol/L)	0.90 ± 0.40	1.18 ± 0.69	<0.001
Potassium (mmol/L)	3.14 ± 0.54	3.29 ± 1.02	0.018
Sodium (mmol/L)	128.34 ± 5.34	126.15 ± 7.07	<0.001
Total bilirubin (umol/L)	70.91 ± 89.27	132.48 ± 134.64	<0.001
Direct bilirubin (umol/L)	55.91 ± 78.73	108.99 ± 110.49	<0.001
Indirect bilirubin (umol/L)	14.29 ± 14.62	23.51 ± 32.32	0.023
Albumin (g/L)	23.73 ± 4.82	21.53 ± 3.95	<0.001
Triglyceride (mmol / L)	4.66 ± 3.00	5.72 ± 3.85	<0.001
Cholesterol (mmol / L)	3.22 ± 2.68	2.75 ± 2.06	0.014
ALT (IU/L)	254.40 ± 340.79	381.82 ± 661.37	0.042
AST (IU/L)	416.12 ± 968.78	1126.30 ± 2213.91	<0.001
LDH (IU/L)	1703.34 ± 2119.80	3745.49 ± 4759.27	<0.001
LDL-C (mmol/L)	2.90 ± 35.15	0.83 ± 1.37	<0.001
LDL-H (mmol/L)	0.60 ± 1.46	0.89 ± 2.08	0.027
APTT (s)	61.03 ± 34.69	78.17 ± 42.04	<0.001
D-dimer (mg/l FEU)	12.94 ± 11.69	15.78 ± 13.02	0.045
Fibrinogen (g / L)	1.24 ± 0.94	1.08 ± 0.79	0.055
PT (s)	21.47 ± 22.75	31.89 ± 31.51	<0.001
PCT (ng/ml)	4.02 ± 8.51	9.21 ± 16.28	<0.001
Urine protein			0.068
0	70 (15.09%)	14 (10.29%)	
+/-	97 (20.91%)	25 (18.38%)	
+	234 (50.43%)	66 (48.53%)	
++	55 (11.85%)	25 (18.38%)	
+++	8 (1.72%)	6 (4.41%)	
Ferritin (ng/ml)			0.211
>2000	388 (83.62%)	122 (89.71%)	
≥500,≤2000	72 (15.52%)	13 (9.56%)	
<500	4 (0.86%)	1 (0.74%)	

AKI, Acute kidney injury; HLH, Hemophagocytic lymphohistiocytosis; DIC, Disseminated intravascular coagulation; PLT, Platelet; HGB, Hemoglobin; WBC, White blood cell; Scr, Serum creatinine; ALT, Aspartate transaminase; AST, Aspartate aminotransferase; LDH, Lactic dehydrogenase; LDL-C, Low density lipoprotein; LDL-H, High density lipoprotein; APTT, Activated partial prothrombin time; PT, Prothrombin time; PCT, Procalcitonin; RRT, Renal replacement therapy.

Risk factors for the development of AKI in patients and Model building

**Figure 2:**
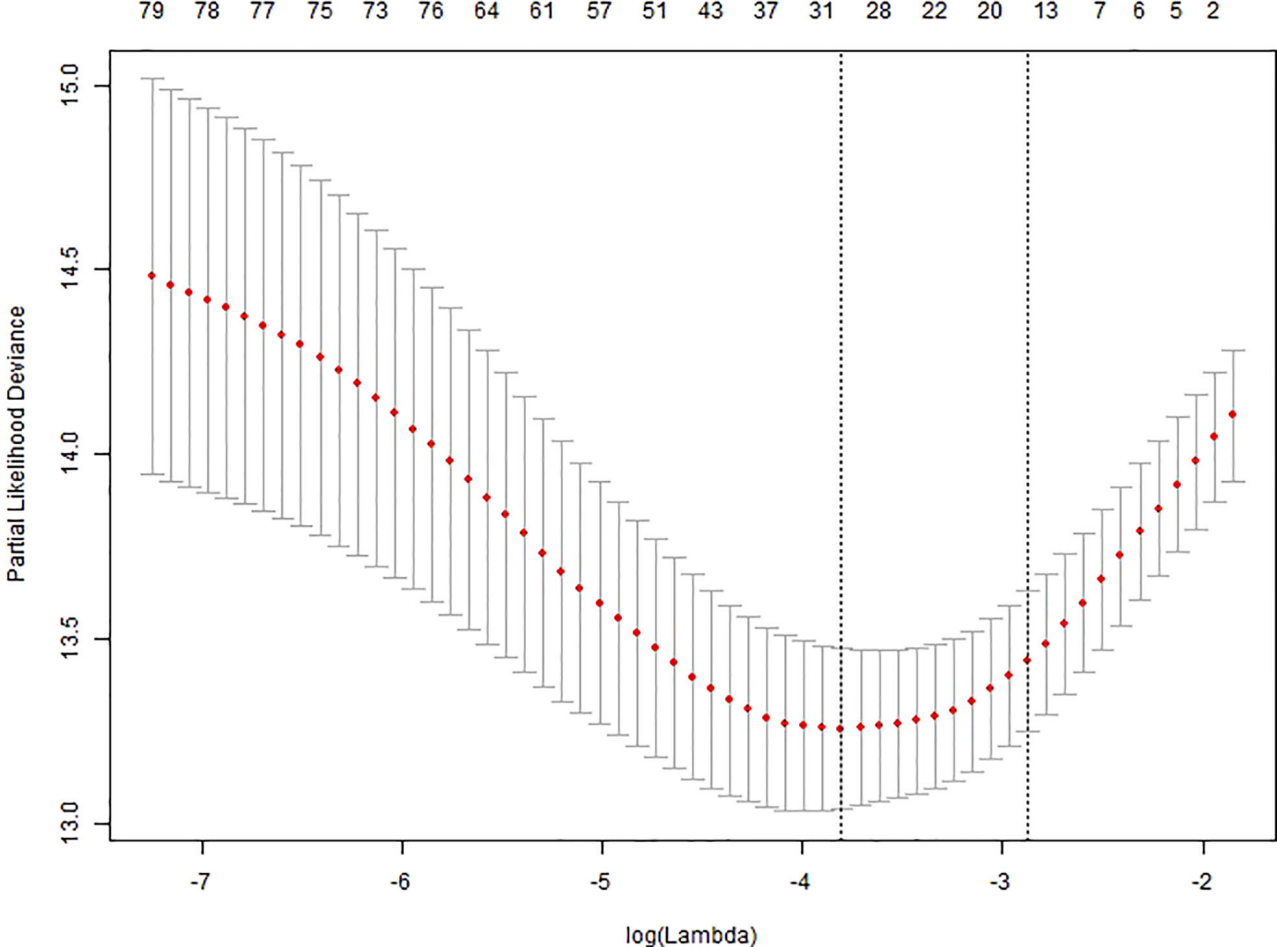
LASSO regression lambda filter graph. The horizontal coordinate is log(lambda) and the vertical coordinate is the mean and standard error of the deviance obtained from Ten‐fold cross‐validation.

**Figure 3:**
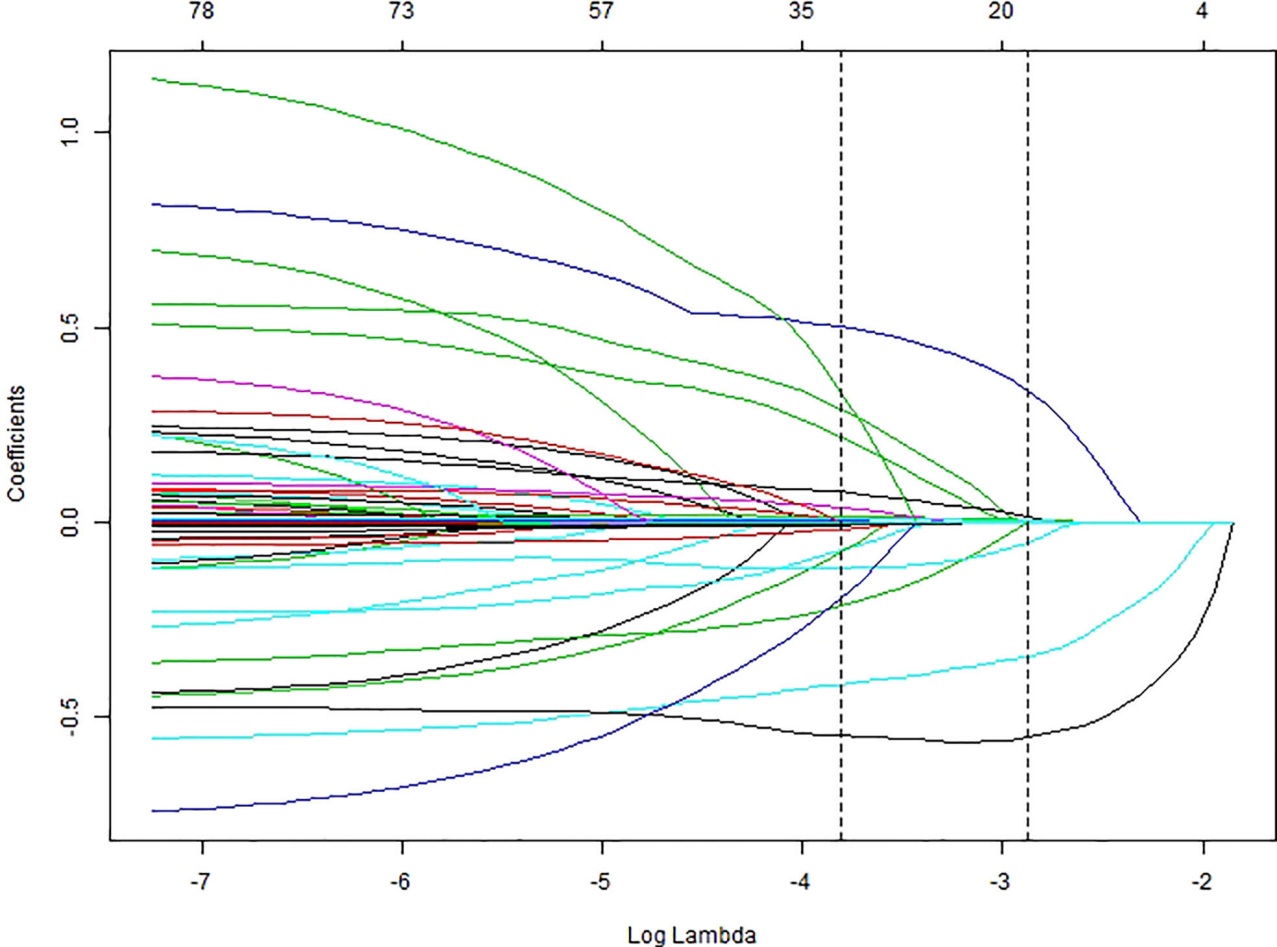
LASSO regression coefficients correspond to lambda values. The bottom scale of the horizontal coordinate is log(lambda), the top scale is the number of variables corresponding to the log(lambda) value, and the vertical coordinate is the lasso regression coefficient.

**Figure 4:**
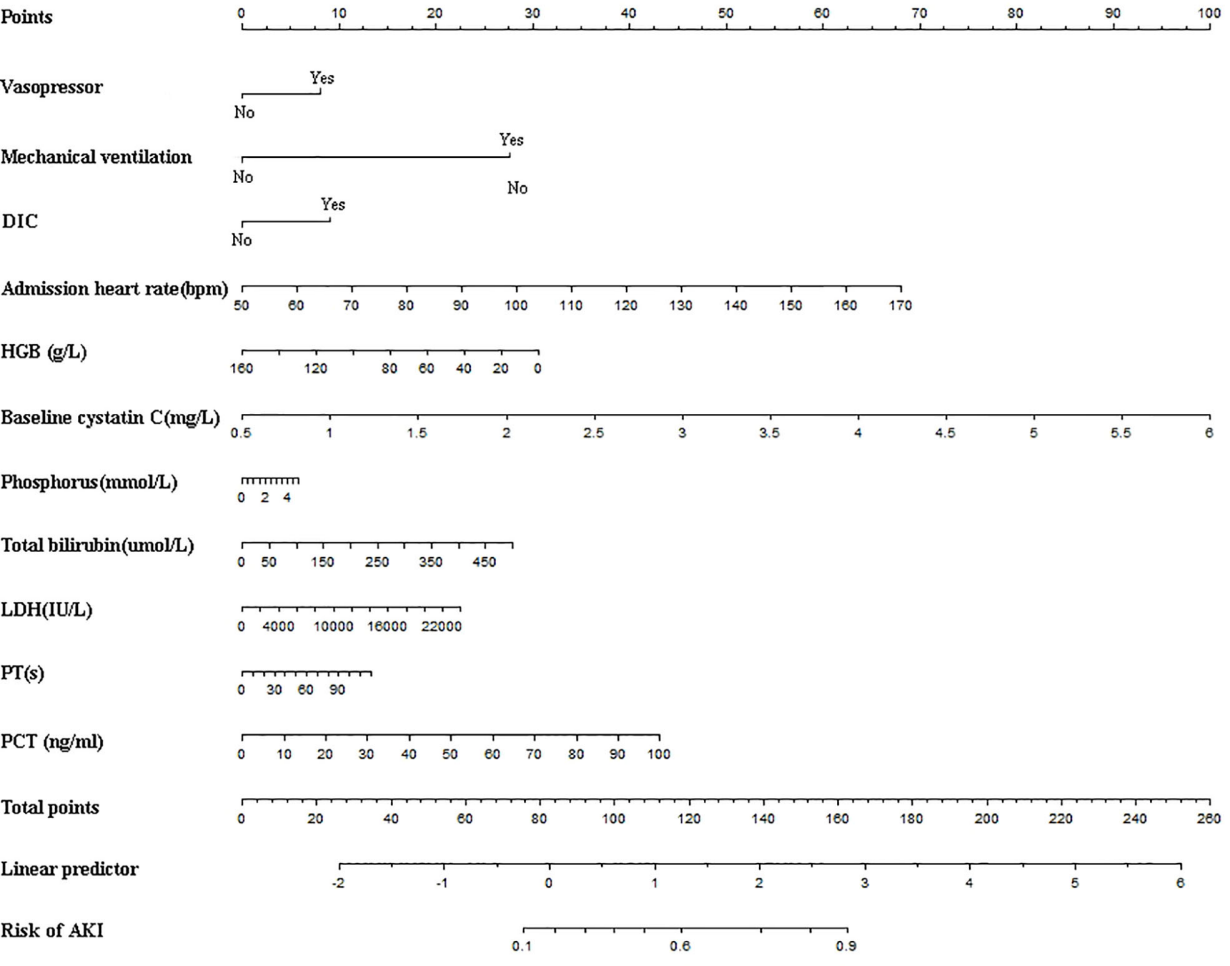
Nomogram for predicting the risk of AKI in patients with HLH. AKI, acute kidney injury; DIC, Disseminated intravascular coagulation; HGB, Hemoglobin; LDH, Lactic dehydrogenase; PT, Prothrombin time; PCT, Procalcitonin.

We performed univariate cox regression analysis on all 600 patients and identified several factors that were highly associated with the occurrence of AKI (**Table 2**). To build the optimal AKI risk prediction model, we conducted LASSO regression analysis of all variables. The LASSO screening variable was calculated based on the maximum lambda corresponding to the selection error mean within 1 standard deviation of the minimum, i.e., lambda = 0.0565 and log(lambda) = -2.8734 (**Figure 2**). Twelve variables were obtained by lasso screening: vasopressor, mechanical ventilation, DIC, admission heart rate, hemoglobin (HGB), baseline cystatin C, phosphorus, total bilirubin, direct bilirubin, LDH, PT, and PCT (**Figure 3**). After covariate screening, direct bilirubin was excluded and the remaining 11 variables were combined to create a risk prediction model for AKI through COX regression. Modeling equations: Logit(Y)=0.28706*(vasopressor=1) +0.97581*(mechanical ventilation=1) +0.31892*(DIC=1) +0.02007*admission heart rate, -0.00676*HGB +0.64382* baseline cystatin C +0.04119*phosphorus +0.00198* total bilirubin +0.00003*LDH +0.00392*PT+0.01527*PCT. The detailed parameters of each variable in the prediction model are shown in **Table 3**. Baseline cystatin C is an independent risk factor for AKI. We then plotted the nomogram based on the results of the COX regression (**Figure 4**).

**Table 2:** Univariate analysis of COX regression in patients with HLH.

	HR (95%CI)	P
Tobacco use (%)	0.85 (0.58-1.25)	0.4105
Alcohol (%)	1.19 (0.83-1.73)	0.3477
Glucocorticoid (%)	0.77 (0.44-1.35)	0.3655
Etoposide (%)	0.63 (0.44-0.89)	0.0090
Nephrotoxic drugs (%)	0.85 (0.60-1.19)	0.3309
Chemotherapy (%)	0.74 (0.51-1.08)	0.1216
Immunosuppressant (%)	0.63 (0.43-0.91)	0.0151
Vasopressor (%)	3.57 (2.55-5.01)	<0.0001
Mechanical ventilation (%)	4.05 (2.87-5.71)	<0.0001
monoclonal antibodies (%)	0.78 (0.40-1.54)	0.4819
Hypertension (%)	1.54 (0.88-2.67)	0.1288
Diabetes (%)	1.08 (0.44-2.63)	0.8711
Heart failure (%)	2.36 (1.47-3.79)	0.0004
DIC (%)	2.79 (1.95-3.98)	<0.0001
Age	1.01 (1.00-1.02)	0.0463
Admission heart rate (Times /min)	1.02 (1.01-1.03)	<0.0001
PLT (×10^9^/L)	0.99 (0.98-0.99)	0.0014
HGB (g/L)	0.98 (0.97-0.99)	<0.0001
WBC (×10^9^/L)	1.03 (0.94-1.13)	0.5784
Baseline Scr (umol/L)	1.01 (1.01-1.01)	<0.0001
Baseline Cystatin C (mg/L)	2.08 (1.63-2.64)	<0.0001
Calcium(mmol/L)	0.37 (0.20-0.68)	0.0014
Phosphorus(mmol/L)	1.81 (1.46-2.25)	<0.0001
Potassium(mmol/L)	1.42 (1.16-1.73)	0.0007
Sodium(mmol/L)	0.96 (0.93-0.98)	<0.0001
Total bilirubin(umol/L)	1.00 (1.00-1.00)	<0.0001
Direct bilirubin(umol/L)	1.00 (1.00-1.01)	<0.0001
Indirect bilirubin(umol/L)	1.01 (1.01-1.02)	<0.0001
Albumin(g/L)	0.92 (0.89-0.96)	<0.0001
Triglyceride (mmol / L)	1.06 (1.02-1.11)	0.0051
Cholesterol (mmol / L)	0.92 (0.84-1.00)	0.0392
ALT (IU/L)	1.00 (1.00-1.00)	0.0054
AST (IU/L)	1.00 (1.00-1.00)	<0.0001
LDH (IU/L)	1.00 (1.00-1.00)	<0.0001
LDL-C (mmol/L)	0.81 (0.70-0.94)	0.0064
LDL-H (mmol/L)	1.08 (0.99-1.17)	0.0981
APTT(s)	1.01 (1.01-1.01)	<0.0001
D-dimer (mg/l FEU)	1.01 (1.00-1.03)	0.0363
Fibrinogen (g / L)	0.82 (0.66-1.03)	0.0829
PT(s)	1.01 (1.01-1.02)	<0.0001
PCT (ng/ml)	1.02 (1.01-1.03)	<0.0001

HLH, Hemophagocytic lymphohistiocytosis; DIC, Disseminated intravascular coagulation; PLT, Platelet; HGB, Hemoglobin; WBC, White blood cell; Scr, Serum creatinine; ALT, Aspartate transaminase; AST, Aspartate aminotransferase; LDH, Lactic dehydrogenase; LDL-C, Low density lipoprotein; LDL-H, High density lipoprotein; APTT, Activated partial prothrombin time; PT, Prothrombin time; PCT, Procalcitonin.

**Table 3:** Multivariate COX regression analysis of variables selected with LASSO for predicting AKI.

Variables	β	SE	HR	Lower 95%CI	Upper 95%CI	P
Vasopressor	0.2871	0.2968	1.3325	0.7448	2.384	0.3335
Mechanical ventilation	0.9758	0.299	2.6533	1.4766	4.7679	0.0011
DIC	0.3189	0.2745	1.3756	0.8033	2.3557	0.2452
Admission heart rate (Times /min)	0.0201	0.006	1.0203	1.0083	1.0324	0.0008
HGB (g/L)	-0.0068	0.0068	0.9933	0.9801	1.0066	0.3198
Baseline cystatin C(mg/L)	0.6438	0.1413	1.9037	1.4432	2.5112	<0.0001
Phosphorus(mmol/L)	0.0412	0.1549	1.0421	0.7692	1.4118	0.7903
Total bilirubin(umol/L)	0.002	0.001	1.002	1.0001	1.0039	0.0418
LDH(IU/L)	0	0	1	1	1.0001	0.2436
PT(s)	0.0039	0.0043	1.0039	0.9956	1.0123	0.3576
PCT (ng/ml)	0.0153	0.0084	1.0154	0.9988	1.0323	0.0697

DIC, Disseminated intravascular coagulation; HGB, Hemoglobin; LDH, Lactic dehydrogenase; PT, Prothrombin time; PCT, Procalcitonin; SE, Standard error; HR, Hazard ratio; CI, Confidence interval.

### Model evaluation

**Figure 5:**
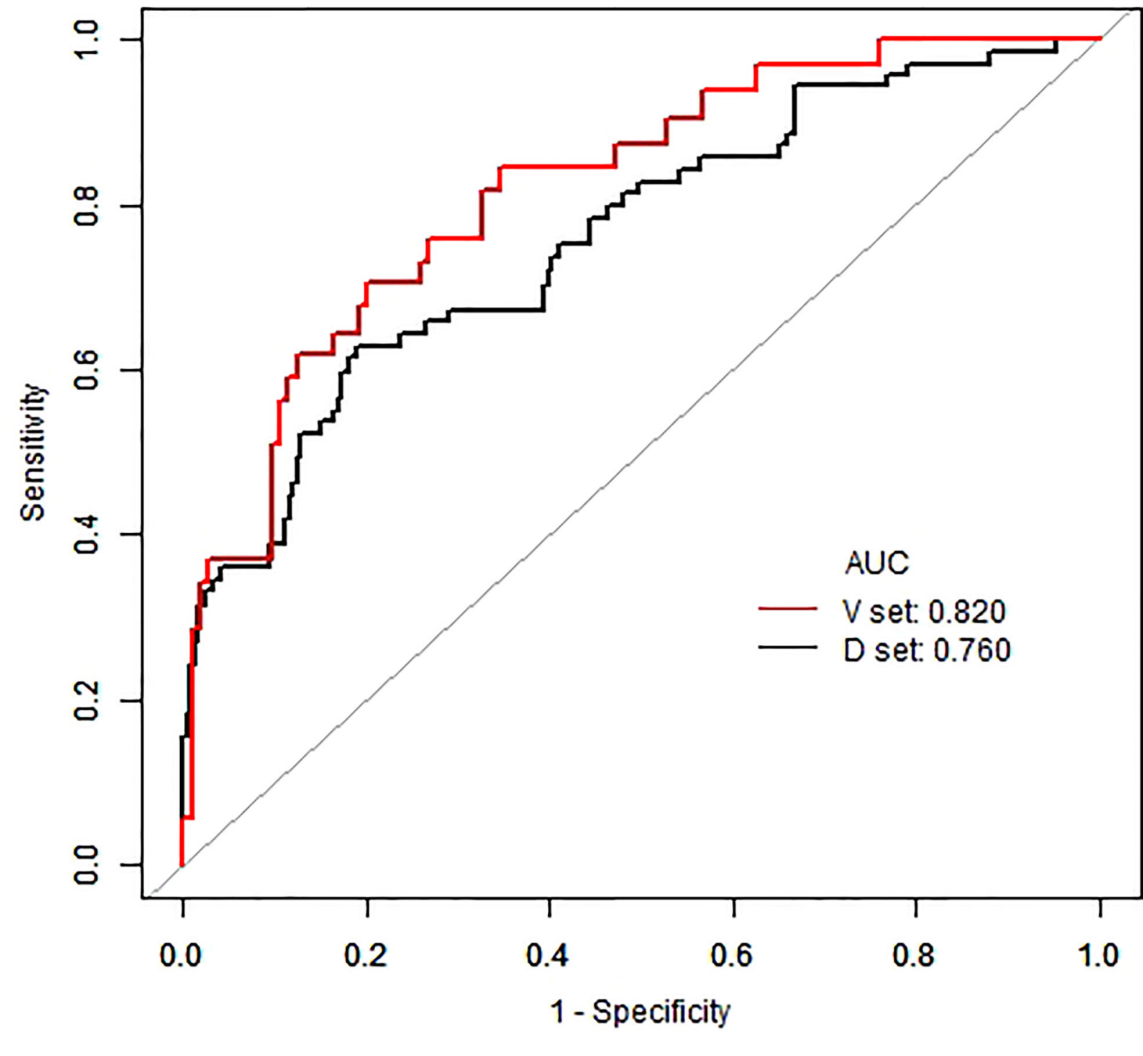
The ROC curves of development and validation set.

The model exhibited good predictive ability with AUC =0.760 for the development group and AUC =0.820 for the validation group (**Figure 5**). C-indexes were calculated in both the development (0.743) and validation set (0.810), indicating the reasonable accuracy of this model. The development group with baseline cystatin C had 4.2% higher predictive power compared with the one without baseline cystatin C (IDI=0.0420, 95%CI 0.0090~ 0.0780, P=0.0070). The validation group model prediction capability also improved by 7.3% (IDI=0.0730, 95%CI 0.0080~0.1570, P=0.0130).

## Discussion

We derived and internally validated a predictive model for the development of AKI in patients hospitalized with HLH. Clinical prediction models (also ‘risk scores’) predict the incidence of a specific disease or mortality by combining multiple risk factors. Risk scores for a multitude of diseases were already clinically applicable to risk-stratify patients and guide treatments (14). Although some shortcomings, there were many other predictive models for secondary AKI that contributed to the improvement of the outcome of AKI patients to some extent (15, 16).

AKI is a clinical syndrome that is susceptible to multiple critical illnesses and usually signals a poor prognosis for the patients. Susantitaphong et al. investigated and found the prevalence of AKI in adults to be about 21.6% (17), which is consistent with our study (22.7%). However, sometimes AKI has an insidious clinical onset and there are no reliable diagnostic and prognostic markers for AKI in patients with HLH at present (2). The delay in AKI diagnosis worsens the severity and prognosis of AKI. Our predictive model can assist physicians in diagnosing AKI on time.

In our prediction model, patients with poor baseline renal function, rapid heart rate on admission, complicated DIC, lower hemoglobin, higher total bilirubin, LDH and PCT, serum phosphorus abnormalities, PT extension, needing for vasopressor and mechanical ventilation were at high risk of AKI. Patients with fast heart rates were more likely to experience AKI (HR 1.0203, 95%CI 1.0083-1.0324, P=0.0008), which has also been identified and applied to the model by other investigators (18, 19). This may be due to the effects of tachycardia on cardiac function and cardiac output, and consequently a decrement in renal perfusion levels (20). Baseline cystatin C was an independent risk factor for the development of AKI (HR 1.9037, 95%CI 1.4432-2.5112, P<0.0001) and enhanced the predictive power of the model. Previous studies have revealed that patients with underlying renal insufficiency tend to have a higher risk of AKI (5, 21), and our study also supported this opinion. Oxidative stress, mitochondrial dysfunction, inflammatory response, vascular dysfunction and other pathological changes in CKD contribute to the heightened sensitivity of AKI (22).

Mild elevation of serum bilirubin protects against renal tubular injury *via* inhibition of oxidative stress and apoptosis, thereby protecting renal function (23, 24). By contrast, high levels of serum bilirubin lead to a lowering of arterial pulse pressure and intraglomerular pressure, exerting direct toxic effects on renal tubule (25–27). Total bilirubin only protects the kidney at <1.2 mg/dl (20.52 umol/L) and promotes the progression of AKI at >2.0 mg/dl (34.20 umol/L) (28). Serum total bilirubin was 84.86 ± 104.45 umol/L, and an independent risk factor for AKI (HR 1.002, 95%CI 1.0001-1.0039, P=0.0418), which is consistent with previous studies. Mechanical ventilation was associated with an elevated risk of AKI in patients (HR 2.6533, 95%CI 1.4766-4.7679, P=0.0011). The pathological mechanism is compatible with mechanical ventilation enhancing the action of inflammatory mediators, leading to epithelial cell apoptosis in the kidney and causing renal dysfunction (29).

To the best of our knowledge, this is the first prediction model for AKI in patients with HLH. This proposed study used LASSO regression to check the variables, and then based on COX regression modeling with different threshold values assigned to each variable. The design gives us the ability to evaluate the weighting of different variables in the diagnosis of AKI. The variables in our prediction model are common and easily accessible in clinical work, and the scoring system is simple and quick, making it easy for clinicians to quickly determine a patient’s risk of AKI and take timely countermeasures. The strong predictive capability and accuracy of the model help us limit misdiagnosis and underdiagnosis.

This prediction model has some drawbacks as well. First, owing to the lack of urine volume, we diagnosed AKI based only on the level of creatinine change. Some patients suffering from AKI before admission may have been omitted. These may lead to underestimation of the incidence rates of AKI. Second, our study could not conclusively determine the cause of AKI occurrence. Retrospective cohort studies may be potentially subject to selection bias. Data for this study were obtained from a single center. Finally, our model was not externally validated, and in the future, we need to perform this work and assess its predictive power with long-term observation.

In conclusion, we developed a risk prediction model for AKI in HLH patients within 28 days of hospitalization. The model applies 11 predictive factors to stratify the risk of AKI occurrence. The predictive power and accuracy of the model are good, and the clinical application is convenient. These may facilitate closer monitoring and early treatment to prevent patients at imminent risk of AKI and heighten clinicians’ alertness to patients at high risk of AKI.

## Data availability statement

The datasets presented in this article are not readily available because the datasets were obtained from the database of West China Hospital and are available from the corresponding author on reasonable request. Requests to access the datasets should be directed to JZ, zhoujiaojiao@wchscu.cn.

## Ethics statement

The studies involving human participants were reviewed and approved by the ethical committee of West China Hospital, Sichuan University. Written informed consent for participation was not required for this study in accordance with the national legislation and the institutional requirements.

## Author contributions

SW and JZ formulated the experimental protocol. The authors SW, JY, XW, and XC collected and compiled relevant data. SW and JZ performed statistical analysis. SW wrote the manuscript and LY, JZ, and LJ revised and commented on the draft. LY and JZ supervised the whole process. JZ provided valuable advice and acquired funding. All authors contributed to the article and approved the submitted version.

## Funding

This study was supported by grants from the National Natural Science Foundation of China [NO. 82102067] and 1·3·5 Project for Disciplines of Excellence–Clinical Research Incubation Project, West China Hospital, Sichuan University [NO. 2020HXFH049], Sichuan, China.

## Acknowledgments

The authors are grateful to the West China Hospital of Sichuan University database for providing open access. We thank Spandidos Publications for the help in language polishing.

## Conflict of interest

The authors declare that the research was conducted in the absence of any commercial or financial relationships that could be construed as a potential conflict of interest.

## Publisher’s note

All claims expressed in this article are solely those of the authors and do not necessarily represent those of their affiliated organizations, or those of the publisher, the editors and the reviewers. Any product that may be evaluated in this article, or claim that may be made by its manufacturer, is not guaranteed or endorsed by the publisher.
